# Impact of Lumbar Postures on the Functioning of Pelvic Floor Muscles Among Osteoporotic Post-Menopausal Women

**DOI:** 10.7759/cureus.32869

**Published:** 2022-12-23

**Authors:** Bhumika Chhibber, Jasobanta Sethi, Harvinder Singh Chhabra, Ankit Jain

**Affiliations:** 1 Department of Physiotherapy, Amity Institute of Health Allied Sciences, Amity University, Noida, IND; 2 Department of Rehabilitation, Indian Spinal Injuries Center, New Delhi, IND; 3 Department of Spine Surgery, Indian Spinal Injuries Center, New Delhi, IND

**Keywords:** hyperlordosis, hypolordosis, vaginal pressure, rectus abdominis, pelvic floor function, osteoporosis

## Abstract

Introduction

Maintaining continence and providing support to the abdominal contents and sexual functioning are among the primary roles of pelvic floor muscles. The pelvic floor muscles work in synergy with the abdominal muscle to perform these functions. Abdominal muscle activation in the sagittal plane is influenced by the lumbar spine posture. As pelvic floor dysfunction is common among post-menopausal women, this study aims to find out the relationship between lumbar posture and electromyographic (EMG) activity of the rectus abdominis (RA) muscle and vaginal pressure (VP) as a functioning of the pelvic floor muscles among osteoporotic post-menopausal females.

Methods

A total of 78 osteoporotic post-menopausal women were recruited and allocated into three groups depending on lumbar lordotic angle, namely normal lordosis (n=26) hyperlordosis (n=26), and hypolordosis (n=26). All the subjects were recorded for RA EMG activity and VP for pelvic floor function in the quiet standing (QS) position, and voluntary dynamic tasks such as maximal coughing (MC) and Valsalva maneuver (VM). Data were analyzed using one-way analysis of variance (ANOVA) and post hoc analysis. A 5% probability level was considered statistically significant, i.e., p<0.05.

Results

The results showed a significant reduction in the RA activity and VP during the dynamic tasks (MC and VM) among subjects with the altered lumbar lordotic angle (p<0.05). The reduction in RA activity and VP was found to be significantly higher (p<0.05) in subjects with hyperlordotic lumbar spine than in those with hypolordotic lumbar spine as compared to normal lordosis during QS, MC, and VM.

Conclusion

We conclude that osteoporotic post-menopausal women with different lumbar lordotic angle show variations in RA activity and pressure generated by the vagina as a function of the pelvic floor during voluntary dynamic tasks.

## Introduction

Maintaining continence and providing support to the abdominal contents and sexual functioning are among the primary roles of pelvic floor muscles (PFMs). Pelvic organ prolapse, urinary incontinence, and fecal incontinence are the conditions included under the umbrella term pelvic floor dysfunction [1]. These disorders lead to a significant proportion of social and economic burdens in the aging population [2]. Ligaments, endopelvic fascia, and connective tissues provide support to the pelvic organs. Various factors such as pelvic trauma, childbirth, lifestyle-related factors, and pregnancy can be major contributing factors to the weakening and detachment of the endo-pelvic fascia in post-menopausal women or the aging population [3].

Osteoporosis has been associated to cause a reduction in bony mass and micro-architectural degeneration of bony tissues [4]. This structural age-related bone mass alteration increases with aging. An exponential increase in the incidences of osteoporosis has been seen with increased life expectancy. A study conducted on 50 Indian females aged 50 years and above shows that 20% of India’s 230 million female population has osteoporosis, while the estimated ratio for Indian men above the age of 50 years stands around 8.5% [5]. Vitamin D deficiency, smoking, alcohol consumption, early menopause, physical inactivity, genetic predisposition, poor eating, and nutritional practices have contributed to osteoporosis among females living urbanized sedentary lifestyles [6]. Multiple causes have been identified. The reversible causes of osteoporosis are calcium deficiency, vitamin D deficiency, decreased sun exposure, and estrogen deficiency in post-menopausal women, while non-reversible causes are gender, genetics, ethnicity, age, and certain metabolic diseases such as hyperparathyroidism [7]. In addition to the above characteristics of osteoporosis, many studies have investigated the association of osteoporosis with spinal stability and abdominal muscle functioning. These studies underlined the reduced strength of trunk muscles among osteoporotic women with different spinal angles in the sagittal plane [8-13]. Change in lumbar lordosis might have been the cause of low bone mineral density [14].

While there have been studies that have very well explained the role of PFMs in spinal stability, this stability needs to co-exist with the synergies between abdominal and PFMs [15]. The capacity of PFMs to contract efficiently may be affected by lumbo-pelvic position. Numerous research studies have looked at how shifting one's bodily posture, such as from lying down to standing, affects PFMs' tonic activity [16]. According to a study, more PFM activity was seen during voluntary PFM contractions made when sitting upright and unsupported as opposed to slumped and supported [17]. It has been found in many studies that sagittal alignment influences the distribution of loads on spinal tissues. The degree of lumbar curvature has been found to cause variations in PFM activity in healthy women [18]. It has been underlined in several studies that normal curvature and an accurately configured spine allow for the systematic contraction of PFMs and potentiate the protection of the pelvic floor from direct intra-abdominal forces [18,19]. Previous literature does not clearly depict the relationship between osteoporosis, posture, and pelvic floor musculature. In this study, we examined the impact of lumbar posture on PFMs functioning in certain activities that are part of daily life.

## Materials and methods

A single-sample, cross-sectional, observational study design was used. A total of 367 females were screened, out of which 78 subjects were recruited from Indian Spinal Injury Centre, New Delhi, India. All the subjects were allocated to three groups based on the lumbar lordotic angle, namely normal curvature (angle between 40° and 70°), hyperlordosis (angle >70°), and hypolordosis (angle <40°). Before undertaking any procedure, all the subjects were explained the safety procedures thoroughly, and written consent was obtained from them.

As this study focused on finding the pelvic floor function altered among osteoporotic post-menopausal women with different lumbar lordotic angles, females with primary osteoporosis, achieved menopause at least two years ago, having body mass index (BMI) between 18.5 to 24.9, and biparous or uniparous with a history of only vaginal delivery were included. This study excluded females with a history of spinal surgery, scoliosis, incontinence (ICQS score >0), nulliparous, hysterectomy, abdominal girth measuring more than 35 inches, a history of low back pain greater than 4 on the numeric pain rating scale, history of any medications for hormones thyroidism in the past one year, any neurological disease that affect balance, any musculoskeletal disease with deformity of the lower limbs, or malignant neoplasia. Participants who fit into the inclusion criteria and did not fit into the exclusion criteria were included and assessed in a structured format.

Each female was recorded for the electromyographic (EMG) activity of the rectus abdominis (RA) along with vaginal pressure (VP) for pelvic floor function during quiet standing (QS), and voluntary dynamic tasks like maximal coughing (MC) and the Valsalva maneuver (VM). All the tasks were performed in the standing position, and each task was repeated three times. The mean value of three repetitions was considered the final value. Each task was done for 5 seconds with a 60-second rest between each task. The pressure exerted by PFMs during the three tasks was assessed by a vaginal probe placed inside the vagina during the performance of these tasks. An Enraf Nonius Myomed 632 X (Enraf Nonius, Rotterdam, the Netherlands) was used along with a vaginal manometer for measuring VP.

Before the experiment began, the placement of EMG electrodes was done bilaterally over RA. Adhesive carbon electrodes with a surface area of 10 mm2 were used. To record the VP, subjects were shown how to use the probe and asked to empty their bladders to standardize bladder volume. The participants were then put in an isolated room with standardized verbal instructions on how to insert a vaginal probe within their vagina. The experimenters used visual inspection to ensure that the probe was properly positioned as coughing and voluntary contractions should cause cranial movement of the probe.

All the statistical analysis was performed using Statistical Package for the Social Sciences (SPSS) Version 20 (IBM Corp., Armonk, NY). Categorical variables were presented in numbers, and continuous variables were presented as mean ± standard deviation (SD). The normality of data was tested using the Kolmogorov-Smirnov test. One-way analysis of variance (ANOVA) test and post hoc analysis were performed to compare quantitative variables. Statistical significance was set at a probability threshold of 5%, i.e., p<0.05.

This study was conducted with ethics approval from Amity University, Noida, Uttar Pradesh, India, and Indian Spinal Injuries Centre, New Delhi.

## Results

All female subjects considered for this study were between 48 and 60 years of age. Groupwise subject demographics have been summarized in Table 1 for age, height, weight, and BMI. The mean and SD of the three groups did not show much difference in all the criteria. There were no missing records in the baseline data for the three groups.

**Table 1:** Demographic details The table explains group-wise demographic data for age, weight, height, and BMI. *Total number of participants included in the study BMI, body mass index

Variable	Groups	n = 78*		Mean	Standard Deviation
		Lower Limit	Upper Limit		
Age (years)	Normal curvature	49	59	55.35	3.11
	Hyperlordosis	48	60	55.15	3.99
	Hypolordosis	48	60	57.04	3.19
Height (centimeters)	Normal curvature	147	170	157.3	6.49
	Hyperlordosis	147	168	155.37	5.4
	Hypolordosis	147	165	154.98	4.93
Weight (kilograms)	Normal curvature	45.1	68.5	57.07	5.66
	Hyperlordosis	45.2	66.5	55.88	5.33
	Hypolordosis	45.4	64	56.48	4.89
BMI	Normal curvature	19.734	24.767	23.03	1.36
	Hyperlordosis	19.7	24.88	23.11	1.39
	Hypolordosis	21.01	24.84	23.48	1.12

As shown in Table 2, significant differences (p<0.05) were found in the EMG activity of RA during MC and VM between all three groups. In contrast, there was no significant difference (p>0.05) found in the EMG activity of RA during QS. Significant differences (p<0.05) were also found in the VP during QS, MC, and VM among all three groups having a different lumbar lordotic angle.

**Table 2:** Between-group analysis (one-way ANOVA) of EMG activity of rectus abdominis, and vaginal pressure recorded during quiet standing, maximal coughing, and Valsalva maneuver in all three groups The table presents a group-wise comparison of mean, standard deviation, standard error, F value, and significance level of rectus abdominis EMG activity, and vaginal pressure data during quiet standing, maximal coughing, and Valsalva maneuver *n=number of participants ANOVA, analysis of variance; EMG, elctromyography; MUAP, motor unit action potential; SD, standard deviation

Variable	Normal Curvature (n=26)*	Hyperlordosis (n=26)*	Hypolordosis (n=26)*	F	Significance
EMG activity (MUAP) in mV of rectus abdominis during quiet standing					
Mean±SD	0.0405±0.00607	0.0213±0.00395	0.0538±0.11158	1.666	0.196
Standard error	0.00119	0.00077	0.02188		
Vaginal pressure (cm of H_2_O) during quiet standing					
Mean±SD	2.1615±0.68473	0.695±0.11615	1.3362±0.54214	54.311	0.000
Standard error	0.13429	0.02278	0.10632		
EMG activity (MUAP) in mV of rectus abdominis during maximal coughing					
Mean±SD	0.8865±0.03878	0.4231±0.04806	0.7273±0.05848	597.886	0.000
Standard error	0.0076	0.00943	0.01147		
Vaginal pressure (cm of H_2_O) during maximal coughing					
Mean±SD	13.0885±4.3213	8.0269±1.33493	11.2423±4.02951	13.947	0.000
Standard error	0.84746	0.2618	0.79025		
EMG activity (MUAP) in mV of rectus abdominis during Valsalva maneuver					
Mean±SD	0.4485±0.2257	0.2238±0.2483	0.3446±0.03962	365.66	0.000
Standard error	0.00443	0.00487	0.00777		
Vaginal pressure (cm of H_2_O) during Valsalva maneuver					
Mean±SD	6.7846±2.15716	3.2423±0.82749	5.0077±1.91727	21.145	0.000
Standard error	0.42305	0.16228	0.37601		

Between-group post hoc analysis presented in Table 3 shows a significant difference (p<0.05) in EMG activity of the RA during MC and VM among all three paired group comparisons. A significant difference (p<0.05) was also found in VP during QS, MC, and VM among all three paired group comparisons.

**Table 3:** Between paired group analysis (post hoc) of EMG activity of rectus abdominis and vaginal pressure recorded during quiet standing, maximal coughing, and Valsalva maneuver across all three groups The table presents the paired group comparison of rectus abdominis activity and vaginal pressure during quiet standing, maximal coughing, and Valsalva maneuver. EMG, electromyography; MUAP, motor unit action potential

Variable	Group Pairing	Mean Difference Between the Two Groups	Standard Error	Significance
EMG activity (MUAP) in mV of rectus abdominis during quiet standing	Normal curvature with hyperlordosis	0.019	0.0179	0.537
	Normal curvature with hypolordosis	-0.013	0.0179	0.736
	Hyperlordosis with hypolordosis	-0.325	0.0179	0.171
Vaginal pressure (cm of H_2_O) during quiet standing	Normal curvature with hyperlordosis	1.467	0.1411	0.000
	Normal curvature with hypolordosis	0.825	0.1411	0.000
	Hyperlordosis with hypolordosis	-0.641	0.1411	0.000
EMG activity (MUAP) in mV of rectus abdominis during maximal coughing	Normal curvature with hyperlordosis	0.463	0.1362	0.000
	Normal curvature with hypolordosis	0.159	0.1362	0.000
	Hyperlordosis with hypolordosis	-0.304	0.1362	0.000
Vaginal pressure (cm of H_2_O) during maximal coughing	Normal curvature with hyperlordosis	5.062	0.9700	0.015
	Normal curvature with hypolordosis	1.846	0.9700	0.004
	Hyperlordosis with hypolordosis	-3.215	0.9700	0.000
EMG activity (MUAP) in mV of rectus abdominis during Valsalva maneuver	Normal curvature with hyperlordosis	0.225	0.0083	0.000
	Normal curvature with hypolordosis	0.104	0.0083	0.000
	Hyperlordosis with hypolordosis	-0.121	0.0083	0.000
Vaginal pressure (cm of H_2_O) during Valsalva maneuver	Normal curvature with hyperlordosis	3.542	0.4808	0.000
	Normal curvature with hypolordosis	1.777	0.4808	0.001
	Hyperlordosis with hypolordosis	-1.765	0.4808	0.001

**Figure 1:**
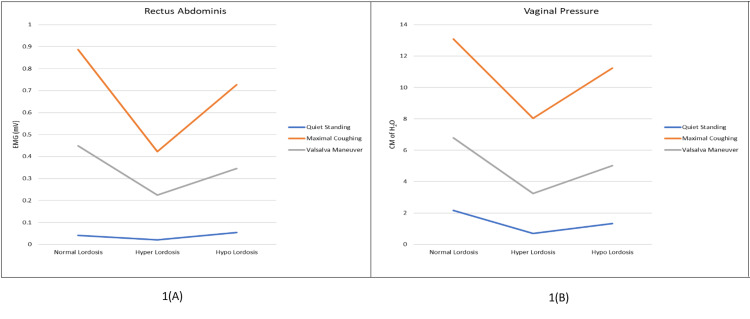
Comparison of the mean value of (A) rectus abdominis activity and (B) vaginal pressure in all three groups with different lumbar lordotic angles during quiet standing, maximal coughing, and Valsalva maneuver EMG, electromyography

Figure 1 shows the comparison of RA activity and VP between different groups having different lumbar lordotic angles during all three tasks. The activity of the RA was found to be reduced as lumbar lordosis changes from normal curvature. There was a more significant reduction in the hyperlordotic group than the hypolordotic as compared to normal curvature. A similar trend was also observed in VP among all three groups. RA activity and VP were found to be highest during MC followed by VM and least during QS.

## Discussion

The aim of this study was to identify the effect of altered lumbar angle on the pelvic floor function generated at rest and while performing the dynamic tasks that challenged both the stability of the lumbar spine and continence systems. In our study, the difference found in the activity of RA among subjects with different lumbar lordotic angles while QS was not significant (p>0.05). At the same time, the activity of the RA was found to be reduced during MC and VM as lumbar curvature deviates from normal in either direction. The reduction of RA activity is found to be more in the hyperlordotic spine as compared to the hypolordotic spine. The results also showed a reduction in resting VP, while this reduction in VP is more in subjects with hyperlordotic spine than hypolordotic during QS. A similar trend of reduction in VP has been observed during MC and VM.

During QS, the no change in the RA activity could be explained by the body adopting anticipatory and compensatory postural adjustments to minimize the postural shift and improve balance while QS. The reduction in the activity of RA among subjects with altered lumbar lordotic angle during dynamic function can be explained as the changes in orientation of the spine resulting in the altered biomechanics of activation of spinal stabilizers. This change in the activity of the spinal stabilizer coexists with the altered lumbar lordosis, resulting in a change in the activation pattern of the RA. Although this activation pattern is time-dependent, the consensus among many researchers is available about a definite change in the activity level of trunk muscle [19]. This finding of our study is contrary to the findings of another study that reported a rise in EMG activity in the rectus muscle in different lumbar postures such as sitting and standing having different lumbar lordotic angles [20]. This study recorded the EMG activity during various voluntary positions adopted, while our study examined EMG activity in normal habitual posture.

In our study, we found a reduction in the pelvic floor function as a lower value of VP during QS, MC, and VM among the subjects with altered lumbar lordotic angle. The higher value of VP among subjects with normal curvature can be understood, as the PFM functions against gravity at their resting length. Since the trunk muscle works synergistically with PFMs to maintain the pelvic floor function, the reduced lumbar lordotic angle in hyperlordotic subjects results in a change in the angle of pull of PFMs, and this change in the angle of pull put extra pressure and inhibits PFMs, which could be the possible reason for the reduced activity in these subjects. While with hypolordotic subjects, prolonged positioning of PFMs in shortened position could have resulted in a reduction in the overall activity of PFMs due to a reduction in the resting tension of PFMs. These findings of our study are also supported by the findings of another study conducted on 16 continent nulliparous women aged between 22 and 41 years who found more PFM activity in habitual lumbar lordosis as compared to hyper- and hypolordosis [21]. At the same time, these findings are contrary to the study demonstrating no association between lumbar angle and pelvic floor dysfunction among 214 elderly women, where the author did not find any correlation between lumbar angle with the presence of pelvic organ dysfunction, although the generalization of dysfunction was limited [22].

As discussed above regarding RA muscle activity and pelvic floor function, it can be stated that the functioning of PFMs gets affected by the lumbar lordotic angle, which, in turn, alters the orientation of muscle fiber and length of the muscle. Although we did not record the changes in the length of the muscles in this study, changes in muscle length may be confirmed in future studies among different lumbar angles. If this length was found to be changed, the study of the change in the length-tension relationship in these muscles can open new dimensions for the researcher to identify the other specific factors associated with pelvic floor functioning and its association with abdominal muscle functioning. In this study, we have seen a marked reduction in pelvic floor function and reduced synergistic activity of the RA in hyperlordotic subjects. Osteoporotic post-menopausal females having hyperlordotic postures are at an increased risk of developing incontinence. Considering this study’s findings, lumbar spine posture can be an important factor responsible for causing alterations in pelvic floor function in osteoporotic post-menopausal women. We also recommend considering lumbar posture correction strategies while addressing the pelvic floor issue among post-menopausal women.

Available literature investigates the trunk muscle activation and pelvic functioning in nulliparous young adults, while this study adds up these findings in menopausal women, who are the larger contributor to the incontinent population. Limitation of this study was that only osteoporotic post-menopausal women were included. We only considered the RA activity, while EMG activities of other trunk muscles and PFMs were not included in this study. We also could not include nulliparous women considering parity as the most common finding among menopausal women. Sexually inactivity can also be one of the contributing factors, which we did not consider in this study. The current literature investigated the activity of trunk muscles while performing dynamic tasks during various consciously adopted postures. This does not clearly explain the effect of habitual posture associated with osteoporosis. Further studies on the length-tension relationship in PFMs associated with different lumbar lordotic angles and time-dependent activation patterns of PFMs can be of future interest.

## Conclusions

From the findings of this study, we conclude that osteoporotic post-menopausal women with different lumbar lordotic angles showed variations in RA activity and pressure generated by the vagina as a function of the pelvic floor during voluntary dynamic tasks. The RA activity and pelvic floor function reduced during voluntary dynamic tasks as lumbar lordosis changed in either direction. This reduction of rectus activity and VP was more in the hyperlordotic spine than in the hypolordotic spine as compared to normal lumbar curvature among osteoporotic post-menopausal women. We also conclude that there was no difference in the activity of the RA during QS among these subjects with variable lumbar lordotic angles.
